# Author Correction: Titers of antibodies against ancestral SARS-CoV-2 correlate with levels of neutralizing antibodies to multiple variants

**DOI:** 10.1038/s41541-023-00600-6

**Published:** 2023-01-25

**Authors:** Trung The Tran, Eline Benno Vaage, Adi Mehta, Adity Chopra, Lisa Tietze, Anette Kolderup, Aina Anthi, Marton König, Gro Nygaard, Andreas Lind, Fredrik Müller, Lise Sofie Nissen-Meyer, Per Magnus, Lill Trogstad, Siri Mjaaland, Arne Søraas, Karsten Midtvedt, Anders Åsberg, Andreas Barratt-Due, Asle W. Medhus, Marte Lie Høivik, Knut Lundin, Randi Fuglaas Karlsen, Reidun Dahle, Karin Danielsson, Kristine Stien Thomassen, Grete Birkeland Kro, Rebecca J. Cox, Fan Zhou, Nina Langeland, Pål Aukrust, Espen Melum, Tone Lise Åvitsland, Kristine Wiencke, Jan Cato Holter, Ludvig A. Munthe, Gunnveig Grødeland, Jan-Terje Andersen, John Torgils Vaage, Fridtjof Lund-Johansen

**Affiliations:** 1 Department of Immunology, Oslo University Hospital, 0424 Oslo, Norway; 2 Department of Pharmacology, Oslo University Hospital, 0424 Oslo, Norway; 3 Institute of Clinical Medicine, University of Oslo, 0315 Oslo, Norway; 4 Department of Neurology, Oslo University Hospital, 0424 Oslo, Norway; 5 Department of Microbiology, Oslo University Hospital, 0424 Oslo, Norway; 6 Division of Epidemiology, Norwegian Institute of Public Health, Oslo, Norway; 7 Division of Method Development and Analytics, Norwegian Institute of Public Health, Oslo, Norway; 8 Division of Infectious Disease Control, Section of Immunology, Norwegian Institute of Public Health, Oslo, Norway; 9 Department of Transplantation Medicine, Oslo University Hospital, 0424 Oslo, Norway; 10 Department of Pharmacy, Oslo University, Oslo, Norway; 11 Division of Emergencies and Critical Care, Oslo University Hospital, 0424 Oslo, Norway; 12 Department of Gastroenterology, Oslo University Hospital, 0424 Oslo, Norway; 13 Influenza Centre, Department of Clinical Science, University of Bergen, Bergen, Norway; 14 Department of Clinical Science, University of Bergen, Bergen, Norway; 15 Section of Clinical Immunology and Infectious Diseases, Oslo University Hospital, Oslo, Norway; 16 Research Institute of Internal Medicine, Sognsvannsveien 20, Oslo University Hospital, Oslo, Norway; 17 Norwegian PSC Research Center, Oslo University Hospital and Institute of Clinical Medicine, University of Oslo, Oslo, Norway; 18 Section of Gastroenterology, Department of Transplantation Medicine, Division of Surgery, Inflammatory Diseases and Transplantation, Oslo University Hospital, Oslo, Norway

**Keywords:** Population screening, Vaccines

Correction to: *npj Vaccines* 10.1038/s41541-022-00586-7, published online 30 December 2022

In this article the author name Marte Lie Høivik was incorrectly written as Marte Lie Høivk. The original article has been corrected.

